# Gut Microbiota and Immunoglobulin A Nephropathy: Exploration of Dietary Intervention and Treatment Strategies

**DOI:** 10.1002/fsn3.70218

**Published:** 2025-05-01

**Authors:** Zhaoyang Dong, Ran Zhang, Liang Shen, Hong‐Fang Ji, Haidong He, Xiaoguo Ji, Liming Zhao

**Affiliations:** ^1^ State Key Laboratory of Bioreactor Engineering East China University of Science and Technology Shanghai China; ^2^ Institute of Food and Drug Research for One Health, School of Food Engineering Ludong University Yantai People's Republic of China; ^3^ Department of Nephrology Minhang Hospital, Fudan University Shanghai China; ^4^ Shanghai Frontiers Science Centre of Optogenetic Techniques for Cell Metabolism, School of Pharmacy East China University of Science and Technology Shanghai China; ^5^ Shanghai Collaborative Innovation Centre for Biomanufacturing Technology (SCICBT) Shanghai China

**Keywords:** diet intervention, gut microbiota, IgAN, treatment of IgAN

## Abstract

Immunoglobulin A nephropathy (IgAN) is a primary glomerular disease characterized by the deposition of IgA. The pathogenesis of it is related to the dysbiosis of gut microbiota. Dysbiosis of gut microbiota influences mucosal immune response and systemic immune system, leading to glycosylation‐deficient IgA1 (Gd‐IgA1) increasing, which promotes the development of IgAN. Diet plays an important role in regulating gut microbiota and treating IgAN. In this review, we summarize the interplay between gut microbiota and IgAN, and their underlying mechanisms. We also describe the effects of dietary intake on IgAN, as well as the composition of gut microbiota. The progress on IgAN treatment mainly focuses on inhibiting or regulating the immune system. Moreover, therapeutic strategies related to gut microbiota such as dietary intervention, supplement of probiotics and prebiotics, as well as fecal microbiota transplantation (FMT) have shown the possibility of improving IgAN prognosis. Thus, exploration of the gut‐kidney axis, the long‐term effects of diet and microbiome is necessary to develop more effective treatment strategies.

## Graphical abstract

This review summarizes the interplay between gut microbiota and IgAN, as well as their underlying mechanisms. We also describe the effect to dietary intake on IgAN and to the composition of gut microbiota.

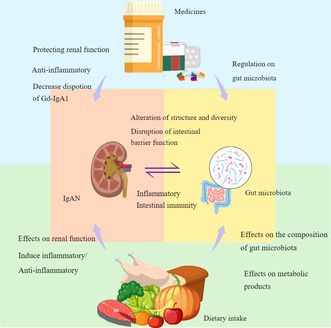

## 1 Introduction

IgAN is a primary glomerular disease first reported by Jacques Berger in 1968. This disease is characterized by the deposition of IgA or IgA predominant immunoglobulin in the mesangial area of the glomerulus (Feehally and Cameron 2011). The symptoms of IgAN could be proteinuria, hematuria, edema, renal dysfunction, and etc. (Kiryluk et al. 2013). According to the report, in 2020, the number of IgAN patients is 9.3 million, and it is expected that patients will reach 10.2 million by 2030 worldwide. IgAN showed different prevalence in different areas, 30%–50% in Asia, 20% in Europe, and 10% in North America (Pattrapornpisut et al. 2021).

IgA is the most abundant immunoglobulin secretion by the mucosal immune system, especially in the gastrointestinal mucosa. Under normal circumstances, IgA produced by the host helps to clear pathogens and maintain mucosal immune homeostasis after infection (Yang and Palm 2020). However, when mucosal infection persists, it can cause abnormal mucosal immune responses, leading to excessive production of IgA1 and resulting in disease. An acknowledged theory is called “multi‐hit hypothesis”: (1) The circulating level of glycosylation‐deficient IgA1 (Gd‐IgA1) increases; (2) Formation of antiglycan immunoglobulin G (IgG) autoantibodies targeted to Gd‐IgA1 formats; (3) Gd‐IgA1, antiglycan IgG autoantibodies, and complement C3 compose a complex; (4) The complexes deposit in the mesangium, causing local inflammation (Pattrapornpisut et al. 2021). Currently, it is considered that the abnormal B cells and plasma cells in the intestinal mucosa of IgAN patients are the main sources of Gd‐IgA (Gesualdo et al. 2021). Meanwhile, dysbiosis of the gut microbiota may lead to damage to the intestinal mucosal, making it easier for Gd‐IgA1 to enter the bloodstream (Cheung et al. 2025). Although current research has revealed multiple key factors, the pathogenesis of IgAN is not fully understood. The treatment of IgAN at present mainly includes supportive therapy, immunosuppression, and target drugs (Kidney Disease: Improving Global Outcomes (KDIGO) Glomerular Diseases Work Group 2021). Some novel drugs are also gradually being discussed for application (Zhi et al. 2022; Liu et al. 2018). However, the treatment of IgAN is still mainly symptomatic treatment, lacking specific treatment plans for the etiology (Kidney Disease: Improving Global Outcomes (KDIGO) Glomerular Diseases Work Group 2021). There are more challenges including the different speed of disease progression and diverse effectiveness of drug treatment among individuals, and side effects and drug resistance caused by long‐term usage of glucocorticoids and immunosuppressants. In that case, more high‐quality clinical trials and exploration of precision medicine strategies are needed in the future. Dysbiosis of the gut microbiota may play an important role in the pathogenesis of IgAN. The gut microbiota significantly impacts the occurrence and development of IgAN by regulating the immune system and mucosal barrier function (Chai et al. 2021; Dong et al. 2022; Han et al. 2022; Tan et al. 2022). The impaired intestinal barrier will increase the permeability of toxins and antigens, activate mucosa‐associated lymphoid tissue, and promote abnormal glycosylation and deposition of IgA1 (Alard et al. 2011). Research has found that the gut microbiota of IgAN patients showed a decrease in beneficial bacteria and an increase in harmful bacteria, which may induce the injury of the intestinal barrier. Additionally, dysbiosis of the gut microbiota may further affect kidney health through changes in metabolites such as shortchain fatty acids (SCFAs). SCFAs not only help maintain intestinal health, but also protect the kidneys by regulating immune and inflammatory responses (Chai et al. 2021). There is a bidirectional relationship between gut microbiota and IgAN, with the “gut‐kidney axis” theory suggesting that gut health directly affects kidney function, and vice versa (Ai et al. 2023; Monteiro and Berthelot 2021). Regulating the balance of gut microbiota, especially increasing the proportion of beneficial bacteria, may become a new direction for the treatment of IgAN.

Diet intake plays an essential role in the management of IgA nephropathy. Some dietary habits are wildly believed with high risk of IgAN. For instance, high protein intake damage glomerular capillary. In Japen, it is obtained that intaking more raw eggs and carbohydrate showed high risk of IgAN (Wakai et al. 2001). Meanwhile, Food allergic and drinking would aggravate IgAN (Wakai et al. 2001). On the other hand, appropriate dietary interventions alleviate symptoms of IgAN, and help IgAN patients to improve. The theory of gut kidney axis reveals the close relationship between gut microbiota imbalance and the occurrence and progression of kidney diseases. This review described the relationship between gut microbiota and IgAN, as well as the research progress on dietary factors, hoping to provide a new perspective for IgAN treatment.

## 2 Gut Microbiota, Immune System and Gut Metabolites in IgAN

Microbiota dysbiosis closely relates to IgAN (Chai et al. 2021; Dong et al. 2022; Han et al. 2022; Tan et al. 2022). Recent research has reported that IgAN patients showed lower microbiota diversity than the healthy control group. By sequencing the 16S ribosomal RNA gene at the V3‐V4 region from IgAN patients and 18 healthy controls, the abundance of Fusobacteria in IgAN patients increased at the phylum level (Hu et al. 2020). Other increased genera, *Escherichia‐Shigella, Hungatella*, and *Eggerthella*, were also found to have pathogenic potential. Moreover, *Bacteroides fragilis, Flavonifractor plautii*, and *Ruminococcus gnavus* enriched in IgAN patients, which were considered characteristic bacteria (Liang et al. 2022). However, *Bifidobacterium* levels decreased in both IgAN patients and IgAN mouse models. The supplementation of *Bifidobacterium* relieved intestinal microbiota imbalance (Tan et al. 2022). The relative abundance of *Prevotella* and *Coprococcus* was also found to be lower in IgAN patients than in healthy controls (Hu et al. 2022). Changes in gut microbiota were reflected in clinical characteristics. For example, estimated glomerular filtration rate (eGFR) is a marker of renal damage. *Clostridium bolteae, Tyzzerella nexilis, Bacteroides vulgatus*, and *Ruminococcus gnavus* displayed a positive correlation with it (Liang et al. 2022), while *Escherichia‐Shigella* was negatively associated with the eGFR (Hu et al. 2020). These findings suggest that altered gut microbiota might be potential biomarkers for IgAN. (The changes gut microbiota abundance Table 1) However, most of the studies were designed as cross‐sectional studies and could not determine the causal relationship between changes in gut microbiota and disease. Moreover, factors that may have a significant impact on gut microbiota, such as the diet and lifestyle of participants, were not recorded in detail, which may interfere with the study results.

**TABLE 1:** The impacts of microbiota alteration to IgAN.

Microbiota and abundance changes	Model	Impact	Potential mechanism	References
Bacteroidetes				
*Bacteroides* ↓	Humanized microbiota‐colonized mice	Exacerbate inflammation in the intestine; promote abnormal deposition of IgA	Promote IgA production through T cell dependent B cell activation pathway	Yang et al. (2020)
Proteobacteria				
*Escherichia‐Shigella*↑	Humanized microbiota‐colonized mice 77 IgAN patients, 66healthy subjects	Exacerbate inflammation in the intestine Increase of plasma Gd‐IgA1	Active TLR4/MyD88/NF—κB signaling pathway; downregulate intestinal tight junction proteins ZO‐1; Humoral response to gut Escherichia‐Shigella result in the increase	Zhu et al. (2024); Gao et al. (2024)
Actinobacteria				
*Bifidobacterium*↓	C57BL/6 mouse model	Exacerbate inflammation; reduce proteinuria	Inhibit NLRP3 and ASC1/Aspase‐1 signaling pathways; reduce the expression of inflammatory factors	Tan et al. (2022)
Firmicutes				
*Faecalibactrium*↓	Transgenic model of mice producing luciferase	Alleviate inflammation in the intestine	Inhibit nuclear factor‐κB(NF‐κB) activation	Breyner et al. (2017)
*Streptococcus*↑	IgAN mice	Exacerbates the inflammatory response of the kidneys; to proliferation of glomerular mesangial cells and matrix deposition Renal biopsy specimens and tonsillar specimens obtained from patients	CCL20/CCR6 pathway activates Th17 cells, and secret of IL‐17 Streptococcus binds to the host extracellular matrix through its surface proteins leading to deposition of Gd IgA	Meng et al. (2014); Ito et al. (2019)
*Lactobacillus*↑	C57BL/6 female mice	Promote abnormal deposition of IgA	Increase the secretion of IgA by the intestine	Mei et al. (2022)
*Coprococcus*↓	Blood and urine specimen from IgAN patients	Promote abnormal deposition of IgA	Affects intestinal barrier function and immune response	Tang et al. (2023)

”↓” represents a decrease in the relative abundance of the flora, and ”↑“ represents an increase of the flora.

The interaction between gut microbiota and the host immune system is described as follows: gut microbiota affects the development and maturation of the immune system, and the immune system in turn shapes the composition of the gut microbiota. The intestinal mucosal immune system develops slowly without microbial interference and is hardly able to resist pathogens. Peyer's patches are small in sterile mice, and the number of immune cells in the lamina propria is low (Zou et al. 2018). The regulation of the immune system by gut microbiota occurs in both innate immunity and acquired immunity (Wang, Ye, et al. 2019; Atarashi et al. 2011; Wong et al. 2017; Thiele Orberg et al. 2017). Specific bacterial species can alter immune responses by promoting the development of certain cells. For example, *Clostridium* specifically induces regulatory T cells and promotes the production of IL‐10 helper T cells in the colon, which are helpful in maintaining immune homeostasis (Atarashi et al. 2011).

**FIGURE 1:**
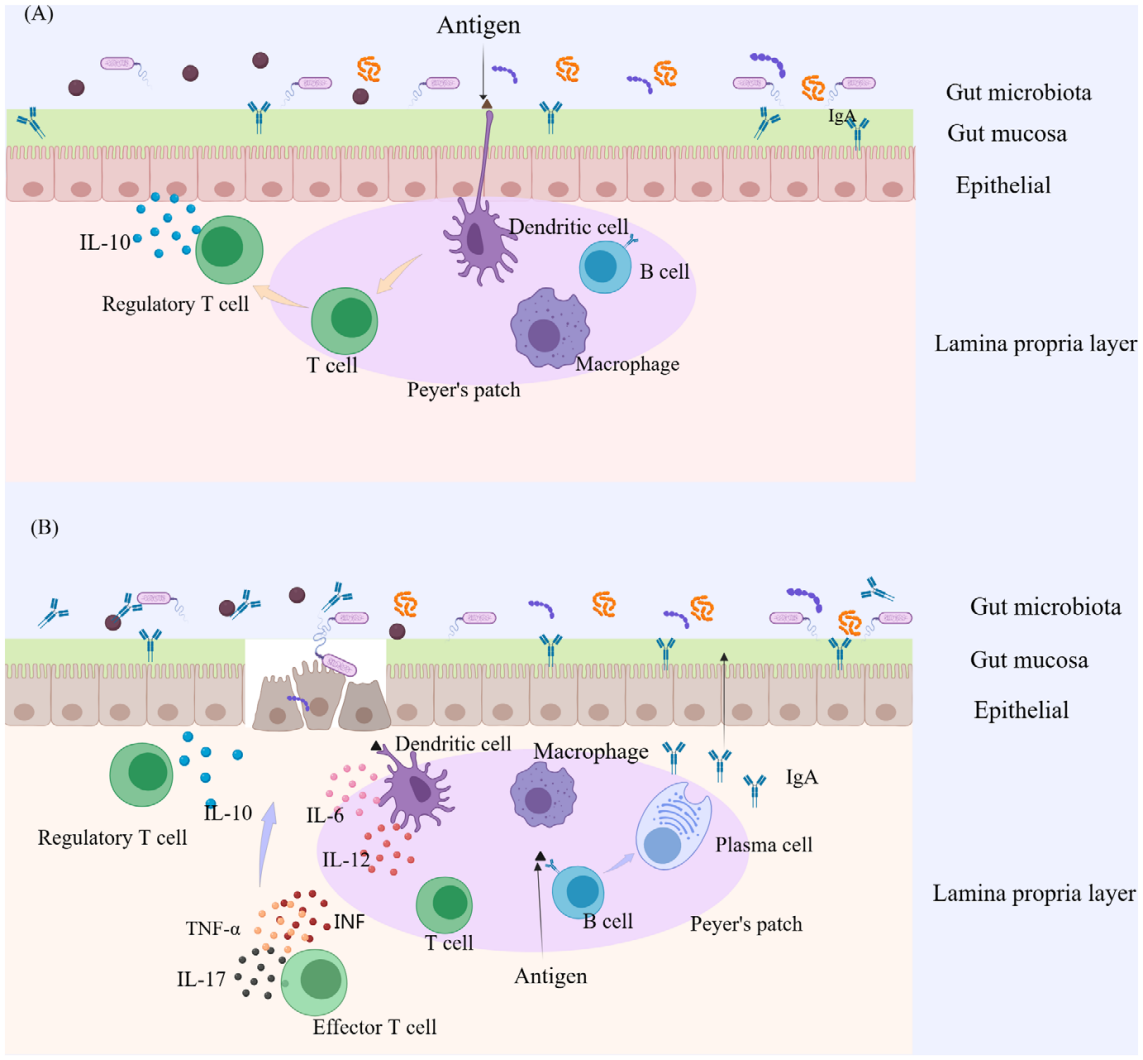
The process of intestinal immunity. The intestinal mucosal barrier is composed of intestinal epithelial cells, a mucus layer, and immune cells, which prevent pathogens and harmful substances from entering the body. The immune cells, including T and B lymphocytes, macrophages, and dendritic cells, embed within the matrix of the payer's patch. (A) When in immune homeostasis, dendritic cells extend dendrites between epithelial cells to sample antigens. The antigens are then broken down and presented to T lymphocytes. These cells are converted into regulatory T cells and migrate to the lamina propria to secrete IL‐10. IL‐10 exerts a suppressive action on immune cells within the lamina propria, so that the gut maintains immune silence, preventing unnecessary inflammation. (B) When the intestinal mucosal barrier is disrupted, the immune cells are activated and regulatory T cells scale down IL‐10 secretion to enable an immune response to proceed. Dendritic cells release inflammatory molecules IL‐6 and IL‐12. Effector T cells migrate to the lamina propria and coordinate an escalation by releasing immune response mediators TNF‐α, interferon (INF) and IL‐17. Meanwhile, B cells are activated and differentiate into plasma cells, which secrete IgA. IgA can neutralize pathogens and toxins, which results in inflammation alleviation.

IgAN is closely related to intestinal immunity and the intestinal barrier. Research has shown that the pathogenesis of IgAN involves mucosal microbiota imbalance, disruption of intestinal barrier function, and immune abnormalities (Wehbi et al. 2019; Wang, Yin, et al. 2019). The intestinal barrier is mainly composed of a mucus layer formed by mucins, tight connections between intestinal epithelial cells, and a microbial barrier formed by the gut microbiota (Kovács et al. 1996; Kloster Smerud et al. 2010). A previous study has shown that *Lactobacillus* can maintain the integrity of the intestinal mucosal barrier. They can, by promoting the secretion of mucin, enhance the thickness and viscosity of the intestinal mucus layer (Zhong et al. 2021). In this case, they prevent pathogens and harmful substances from contacting intestinal epithelial cells. When these beneficial bacteria decrease, the mucus layer becomes thinner and the integrity of the intestinal barrier is disrupted, making it easier for pathogens and harmful substances to penetrate the intestinal wall and trigger inflammatory reactions (Gesualdo et al. 2021). Disruption of gut microbiota, manifested by an increase in pathogenic bacteria and a decrease in beneficial bacteria levels, further affects the progression of IgAN. For example, a study found that Gd‐IgA1 levels increased in untreated IgAN patients correlating with toll‐like receptor 4, B‐cell stimulators, and proinflammatory cytokines. The following experiment colonized mice with gut microbiota from IgAN patients and observed the activation of the TLR4/MyD88/nuclear factor‐κB pathway, as well as IgAN propensity (Zhu et al. 2024). Additionally, Gamma delta T cells (γδT cells) regulate the stability of the intestinal barrier and mucosal immune response by identifying microbiota‐associated molecular patterns produced by the gut microbiota. They are located in the intestinal epithelium and can promote the maintenance of intestinal barrier function by secreting cytokines such as IL‐17 (Zhong et al. 2021). Research has shown that the production of IgA1 is significantly increased in mice with γδT cell deficiency, indicating that γδT cells play an important role in maintaining normal glycosylation and function of IgA1 (Ruszkowski et al. 2018). In an IgAN mouse model infected by streptococcal bacteria, the expression of chemokine ligand 20 (CCL20) increased and recruited more Th17 cells. These processes were inhibited by anti‐CCL20 (Meng et al. 2014), which provided a new target for clinical treatment. In addition, gut microbiota play a crucial role in the production of mucosal Gd‐IgA1 and the development of IgAN (Monteiro and Berthelot 2021; Kiryluk et al. 2014; Sallustio et al. 2021). Lipopolysaccharide activates the toll‐like receptor 4 signaling pathway, inhibits the expression of core 1, β1, 3‐galactotransferase specific molecular companion gene, resulting in IgA1 lacking galactose modification and forming Gd‐IgA1 (Tian and Jian 2023). Regulating and controlling gut microbiota disorders may provide new directions for the treatment of IgAN. The targeted release of budesonide in the intestine achieves therapeutic effects by regulating intestinal immunity, showing that the drug can significantly reduce urinary protein levels in IgAN patients. These research advances suggest that gut microbiota and intestinal mucosal immunity play important roles in the pathogenesis of IgAN and may become new targets for the future treatment of IgAN (Barratt, Lafayette, Kristensen, et al. 2023). (The process of intestinal immunity is shown in Figure 1).

**FIGURE 2:**
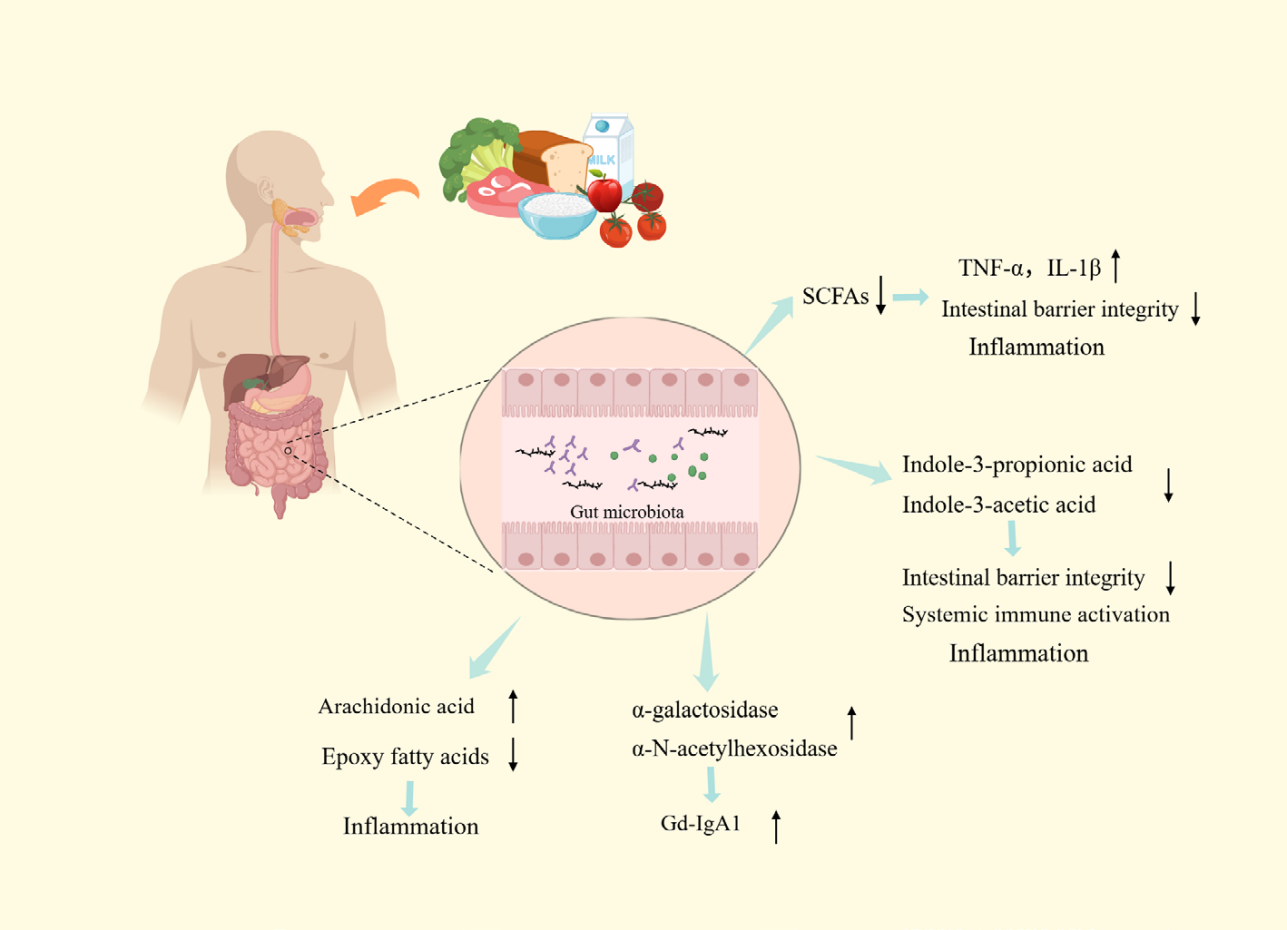
The impact of metabolites on IgAN. Gut metabolites alter in IgAN patients, and these alterations then affect the disease. SCFAs and amino acid metabolites reduce in IgAN patients with inflammation and damage to the intestinal barrier integrity, while arachidonic acid is increased, but also causes inflammation. Enzymes α‐ galactosidase and α‐N‐acetyl galactosidase significantly enriched, resulting in the levels of Gd‐IgA1 increasing. Metabolites aromatic amino acids, such as indole‐3‐propionic acid and indole‐3‐acetic acid, are observed to decline.

The metabolites of gut microbiota are diverse, and the most common ones are SCFAs, aromatic hydrocarbon receptors, and polyamines. These metabolites play an important role in regulating intestinal immunity (Di Leo et al. 2021; Papista et al. 2015). Besides, gut microbiome also affects renal metabolites profile (Claus et al. 2008). Clinical studies have found that the levels of unsaturated fatty acids and fatty acid derivatives in the intestines of IgAN patients are significantly reduced. The protective intestinal metabolites such as prostaglandin derivatives and epoxy fatty acids are declined, while the level of the pro‐inflammatory metabolite arachidonic acid increased, which might be led by abnormal metabolic pathways of linoleic acid and arachidonic acid (Wu, Qian, et al. 2021). Lower SCFAs were measured in IgAN mice than wildtype mice. Moreover, providing IgAN mice extra SCFAs directly led to the decrease of the level of IgA in the kidney tissues, the level of urinary protein, and the levels of serum inflammatory factors TNF–α and IL‐1β (Tan et al. 2022). Noticeably, as the key enzymes of protein glycosylation, α‐ galactosidase and α‐N‐acetylgalactosidase are significantly enriched in IgAN patients. Other enzymes related to the production of Gd‐IgA1, such as β‐galactosidase and β‐N‐acetylhexosidase are also enriched (Liang et al. 2022). Significant metabolic disorders of aromatic amino acids (such as tryptophan) in IgAN patients resulted in the levels of their metabolites (such as indole‐3‐propionic acid and indole‐3‐acetic acid) elevated. In IgAN patients, the decrease in indole‐3‐propionic acid levels led to aggravated inflammation (Wu, Tang, et al. 2021; Li et al. 2021). (The impact of metabolites on IgAN is shown in Figure 2).

## 3 Diet and IgAN

There is a close relationship between diet and physical health. Reasonable eating habits provide essential nutrients for the body, enhance immunity, and prevent diseases, while poor eating habits may lead to health problems such as malnutrition, obesity, cardiovascular disease, diabetes, etc. (Martinez‐Gonzalez and Martin‐Calvo 2016; Kopp 2019; Waddell and Orfila 2023). The distinct compositions of typical diet models affect the intestinal microenvironment and impact IgAN. (How different diet pattern affect IgAN is shown in Table 2).

**TABLE 2:** Dietary intake affects IgAN.

Dietary pattern	Characteristics of dietary pattern	Impact on IgAN	Microbiota changes	References
High‐fat Diet	Rich in animal fats, fried foods, and cream products	Damage renal tubular epithelial cells, change gut microbiota composition	*Firmicutes*↑, *Actinobacteria* ↑*Proteobacteria*↑ *Bacteroides*↓	Kaartinen et al. (2007); Hildebrandt et al. (2009)
High‐fiber Diet	Rich in dietary fiber, such as vegetables, fruits, and whole grains	Relieve inflammation, improve intestinal barrier function, alleviate the pathological progression of IgAN	*Prevotella*↑ *Bifidobacterium*↑ *Lactobacillus*↑ *Faecalibacterium*↑	Costabile et al. (2008); Häger et al. (2019)
High‐protein Diet	Contains large amounts of proteins (such as meat, eggs, legumes)	Increases renal burden, worsen proteinuria, renal insufficiency	*Bacteroides* ↑ *Lacticaseibacillus*↑, *Streptococcus*↑	Aluko et al. (2015); Ma et al. (2017)
Mediterranean Diet	Primarily consists of vegetables, fruits, fish, whole grains, legumes, and olive oil	Rich in antioxidants and unsaturated fatty acids, helps reduce the risk of chronic kidney disease, and may delay renal function decline	*Faecalibacterium*↑ *Ruminococcus*↓	Di Rosa et al. (2023); Jin et al. (2019)
Intermittent Fasting	Have a normal diet for a certain period of time, and restrict or completely fast for another period of time	Increasing the abundance of beneficial bacteria, reducing harmful bacteria, enhancing intestinal immunity	*Bacteroidetes*↑ *Lactobacillus*↑ *Fusobacterium*↓ *Firmicutes*↓	Zeb et al. (2023); van der Merwe et al. (2020)
Gluten free diet	Avoid wheat, barley, rye and their products	Long term gluten free diet may lead to an imbalance in the gut microbiota in healthy humans; increase risk of inflammation; but prove the symptoms and intestinal health of patients with celiac disease	*Enterobacteriaceae*↑ *Bifidobacterium*↓ *Lactobacillus*↓	Wu, Qian, et al. (2021); Sanz (2010)
Alcohol	Adults' daily alcohol intake should < 15 g	Promote inflammatory response; increase immunoglobulin deposition; increase the risk of end‐stage renal disease	*Bacteroidetes*↑*Firmicutes*↑ *Proteobacteria*↓	Hartmann et al. (2013); Bull‐Otterson et al. (2013)
Fermented dairy	Include cheese, yogurt	Increase the risk of IgAN	*Bifidobacterium*↑	Romond et al. (1998); Li et al. (2024)
Salt	Healthy adults daily salt intake should < 5 g	Excessive salt intake may activate the RAS; decreased glomerular filtration rate; exacerbate kidney damage	*Lachnospiraceae*↑ *Ruminococcus*↑ *Lactobacillus*↓	Hu et al. (2017); Miranda et al. (2018)

A high‐fat diet induces abnormal lipid metabolism, characterized by elevated levels of triglycerides and cholesterol. These metabolic changes directly participate in the damage process of renal tubular epithelial cells (Kaartinen et al. 2007). Additionally, a high‐fat diet changes gut microbiota composition. For instance, safflower oil reduces the populations of *Bacteroides* and enriches the populations of *Firmicutes*, *Actinobacteria*, and *Proteobacteria*, which affect the adhesion and growth of gut microbiota (Hildebrandt et al. 2009). A high‐fat diet also affects the expression of tight junction proteins such as Occludin and ZO‐1. A study detected the levels changes of Occludin and ZO‐1 in the intestinal tissue of mice fed a high‐fat diet through Western blot. The results showed that compared with the normal diet group, the protein levels of Occludin and ZO‐1 in the intestinal tissue of mice in the high‐fat diet group were significantly reduced (Yamasaki et al. 2024). The low levels of tight junction proteins induce destruction of the intestinal barrier and cause systemic inflammation.

High fiber diet alters the structure of gut microbiota; for example, polysaccharides promote the growth of beneficial microbiota such as *Bifidobacterium* and *Lactobacillus* while inhibiting harmful flora (Costabile et al. 2008). The fermentation of fibers, such as SCFAs, helps to maintain gut balance. SCFAs lower intestinal pH, an important indicator of gut health, and inhibit the growth of potentially pathogenic bacteria like *Enterobacteriaceae* (den Besten et al. 2013; Zimmer et al. 2012; Ghosh et al. 2011). Moreover, abnormal blood lipids exacerbate kidney damage, and the soluble fiber in high fiber foods is able to decrease them. For instance, β‐glucan and pectin bind with bile acids in the intestine, promoting their excretion and reducing blood cholesterol and triglyceride levels (Luna‐Castillo et al. 2022). In addition to its lipid‐lowering effects, a high‐fiber diet also displays anti‐inflammatory properties. Its intake is inversely proportional to inflammatory markers, such as tumor necrosis factor alpha (TNF—α) and interleukin‐6 (IL‐6) (Häger et al. 2019). Given that IgAN patients generally have a mild inflammatory state, the high fiber diet helps to reduce the levels of these inflammatory factors, alleviate kidney inflammation, and delay the progression of IgAN (Niero et al. 2023; Wagenaar et al. 2021).

Long term high protein diet may increase renal burden and accelerate disease progression. Patients with IgAN usually have impaired kidney function, and a high protein diet increases the burden on the kidneys in metabolizing nitrogen‐containing substances such as urea and uric acid. This leads to the accumulation of these metabolic products, increasing the burden on the kidneys and accelerating the progression of IgAN. Meanwhile, a high protein diet keeps the glomerulus in a state of high filtration and high perfusion, increasing glomerular filtration rate (GFR) (Aluko et al. 2015). A high protein diet also increases the relative abundance of *Bacteroidetes*, which will induce the production of IgA (Neis et al. 2015). However, proteins are considered an essential nutrient for the human body. Despite a high‐protein diet may increase renal burden, the intake of high‐quality protein remains important for IgAN patients.

Intermittent Fasting refers to having a normal diet for a certain period of time and to restrict or completely fast for another period of time. This diet pattern is considered helpful to reduce weight and improve metabolism. Additionally, multiple studies have shown that intermittent fasting can significantly increase the diversity of gut microbiota (Zeb et al. 2023). A study used C57BL/6 male mice, divided into a control group with free feeding and an experimental group with restricted feeding during the last 4 h of the light cycle. All mice received a high‐fat diet (HFD) to investigate the effects of feeding time on obesity‐related parameters and gut microbiota. The alpha diversity of the gut bacterial community in the experimental group mice was significantly higher than that in the control group mice, and the relative abundance of beneficial bacterial genera such as *Lactobacillus*, *Bifidobacteria*, and *Akkermansia* increased significantly (van der Merwe et al. 2020). It increased the relative abundance of beneficial bacteria such as *Bacteroidetes* and *Lactobacillus* and reduced the relative abundance of harmful bacteria such as *Fusobacterium* (Zeb et al. 2023; van der Merwe et al. 2020). However, the sample size of this research is small and lacks long‐term clinical research data. Its safety and long‐term effects still need to be verified through more human clinical trials and long‐term studies. Intermittent fasting also increased intestinal mucosal thickness and villus length in db/db mice, which suggested enhancing the integrity of the intestinal barrier (Guo et al. 2023). Although there is no research showing the impact of intermittent fasting, it positively affects IgAN by reducing inflammatory factors and enhancing intestinal immunity.

The daily diet of IgAN patients not only affects the filtration function of the kidneys, but is also related to inflammatory response, blood pressure control, and overall metabolic health. Research has shown that appropriate dietary adjustments, such as a low protein diet, limiting sodium intake, and increasing dietary fiber, can significantly reduce kidney burden, lower proteinuria, and slow down further deterioration of kidney function (Kramer 2019). Limiting sodium intake is one of the widely applicable rules in the dietary management of IgAN. Research has shown that reducing sodium intake effectively improves patient prognosis, lowers blood pressure, and reduces proteinuria levels. Protein is another nutritional need to be noticed. Although protein is recommended for health in the population, IgAN patients should limit protein intake. A gluten‐free diet is considered to improve IgAN symptoms, especially to alleviate glomerular immune protein disposition and proteinuria (Papista et al. 2015). In addition, specific dietary patterns, such as the Mediterranean diet, are believed to be beneficial for IgAN patients due to their anti‐inflammatory and cardiovascular protective properties (Kramer 2019). However, the individualized needs and long‐term compliance of dietary interventions remain challenges in clinical practice. Therefore, in‐depth exploration of the relationship between diet and IgAN is of great significance for developing effective dietary intervention strategies and improving patient prognosis.

## 4 Research Progress in the Treatment of IgAN

The treatment strategies of IgAN can be divided into three types according to their function: (1): improve renal function; (2) anti‐inflammation; (3) decrease the disposition of Gd‐IgA. (The treatment strategies of IgAN are shown in Table 3).

**TABLE 3:** Progress on IgAN treatment.

Treatment strategies	Medicine	Function	Into use or not	References
RAS	Losartan	Alleviate proteinuria	Marketed	Floege et al. (2021)
Glucocorticoid	Prednisone	Anti‐inflammatory	Marketed	Kidney Disease: Improving Global Outcomes (KDIGO) Glomerular Diseases Work Group (2021)
Immuno‐suppressant	Mycophenolate Mofetil (MMF)	Inhibiting the proliferation of T and B lymphocytes; alleviate proteinuria	Marketed	Hou et al. (2017)
	Felzartamab	Inhibit CD38+ plasma cells secrect Gd‐IgA1 and its antibody; alleviate glomerular damage	Phase II clinical trial	Mayer et al. (2025)
	Nefecon	Inhibit the proliferation of specific bacteria in the intestine, reduce the synthesis of IgA	Marketed	Barratt, Lafayette, Kristensen, et al. (2023); Barratt, Lafayette, Rovin, et al. (2023)
Immunoregulator	Hydroxychloroquine (HCQ)	Immune regulation; anti‐inflammatory; reduce renal damage	Marketed	Liu et al. (2019)
	Bortezomib
	Plasma cells inhibitor; alleviate proteinuria	Marketed	Hartono et al. (2018)
	Iptacopan
	B‐factor inhibitors; inhibit the alternative pathway of the complement pathway	Phase III clinical trial	Perkovic et al. (2024)
Atacicept
Interfere the maturation and differentiation of B cells; reduce Gd‐IgA1 levels	Phase III clinical trial	Lafayette et al. (2024)
Telitacicept
Target B cells to reduce IgA production; reduce Gd‐IgA1 levels; alleviate proteinuria	Phase III clinical trial	Lv et al. (2023)
Novel treatment strategies	Fecal microbiota transplantation (FMT)	Exacerbate inflammation; reduce proteinuria	Clinical trial	Zhi et al. (2022); Zhao et al. (2021)
	Probiotics, prebiotics, and synbiotics	Improve intestinal microbiota imbalance; alleviate proteinuria	Mouse model	Tan et al. (2022)
SGLT2	Dapagliflozin	Alleviate proteinuria; improve renal function	Marketed	Barratt and Floege (2021)
Traditional Chinese medicine	Shenyan Kangfu tablet	Alleviate proteinuria	Marketed	Chen et al. (2021)

Renin‐Angiotensin System (RAS) inhibitors are the most commonly used medications to protect renal function. They significantly reduce urinary protein levels by inhibiting sodium absorption in the proximal tubules and increasing sodium delivery to the distal tubules (Floege et al. 2021). Sodium‐glucose co‐transporter 2 (SGLT2) inhibitors have also been proven effective in clinical trials. SGLT2 located in the proximal tubules of the kidney and it is mainly responsible for glucose reabsorption within the renal element. Its inhibitors inhibit the absorption of sodium by the proximal tubules, increase the delivery of sodium by the distal tubules, activate tubular glomerular feedback, and normalize glomerular filtration rate. The results of SGLT2 inhibitor treatment in chronic kidney disease showed a reduction of the ratio of urinary albumin to creatinine (Barratt and Floege 2021). Both RAS inhibitors and SGLT2 inhibitors have demonstrated the efficacy in improving renal function and reducing proteinuria. These pharmacological interventions are crucial components of the current treatment strategies for IgAN.

Anti‐inflammatory treatment is another component in the management of IgAN. Common medications for anti‐inflammatory purposes include glucocorticoids and immunosuppressants. Glucocorticoids are recommended for IgAN patients with urinary protein ≥ 1 g/d (Kidney Disease: Improving Global Outcomes (KDIGO) Glomerular Diseases Work Group 2021). However, long‐term use of glucocorticoids is associated with numerous adverse reactions, and their benefits in treating high progression risk IgAN are still controversial (Lv et al. 2017). The adverse reactions of glucocorticoids include protein and lipid metabolism, hypertension, hyperglycaemia, as well as osteoporosis (Kidney Disease: Improving Global Outcomes (KDIGO) Glomerular Diseases Work Group 2021). Immunosuppressants, such as mycophenolate mofetil (MMF), are able to inhibit the proliferation of T and B lymphocytes and regulate immune function. A study investigated the efficacy of MMF combined with prednisone versus full‐dose prednisone in 176 IgAN patients with active proliferative lesions. The result illustrated that compared to full‐dose prednisone, the combination of MMF and prednisone failed to reduce proteinuria but resulted in fewer adverse events in IgAN patients with active proliferative lesions (Hou et al. 2017). Although immunosuppressant therapy for IgAN can improve the condition of patients to some extent, the side effects are still significant and diverse. Immunosuppressants may significantly increase the risk of infection by suppressing immune system function. Moreover, long‐term use of immunosuppressants may cause damage to the liver and kidneys (Zhang et al. 2019).

The multi‐hit hypothesis posits that IgAN is caused by the deposition of IgA. Reducing the production of IgA and Gd‐IgA may prevent the disease from the outset. B cells participate in antibody production. By reducing the number of B cells, interfering with their maturation and differentiation, and lowering serum IgA levels, disease progression can be mitigated. The results of a phase II clinical study on the treatment of IgAN with Telitacicept showed that patients receiving Telitacicept treatment had significantly reduced levels of IgA in blood (50.4% lower than the control group), accompanied by an average decrease of about 49% in proteinuria (Lv et al. 2023). Currently, targeted‐release budesonide, which is already on the market, is involved in therapeutic treatment. It is able to slowly release the drug in the distal ileum and proximal colon to resist inflammation, inhibit immune responses, as well as reduce the production of Gd‐IgA1 (Barratt, Lafayette, Rovin, et al. 2023). Clinical trials illustrated its capability to reduce the urine protein creatinine ratio of IgAN patients (Barratt, Lafayette, Kristensen, et al. 2023).

Recent years, novel treatments focusing on regulating gut microbiota are explored (Liu, Song, et al. 2024; Liu, Triffitt, et al. 2024; Chen et al. 2021). Research suggests that the use of probiotics, prebiotics, and symbiotics may regulate the composition, types, and proportions of gut microbiota. According to the relationship between IgAN and gut microbiota mentioned above, it is expected that gut inflammation and changes in gut microbiota might affect the recovery of IgAN. Probiotics regulate immune responses and reduce the production of inflammatory factors. Supplementing probiotics such as *Bifidobacterium* and *Lactobacillus* can improve intestinal microbiota balance. The growth of beneficial bacteria also improves the structure of gut microbiota (Tan et al. 2022). In this case, the intervention of probiotics is involved in the treatment of kidney diseases such as chronic kidney disease and renal insufficiency. Fecal microbiota transplantation (FMT) involves transplanting gut microbiota obtained from healthy donors to the patient's gastrointestinal tract. It has been applied to treat gastrointestinal diseases caused by microorganisms, recurrent *Clostridium difficile* infection, metabolic syndrome and etc. (Antushevich 2020). Several IgAN patients accepted the treatment of FMT and showed relieving clinical symptoms (Zhi et al. 2022; Zhao et al. 2021). The mechanisms of FMT treating IgAN are unclear, but they might be related to increased diversity of gut microbiota. Moreover, a study displayed that FMT alleviated experimental colitis in mice with increased IL‐10 secretion by activating specific immune cells, such as CD4+ T cells and antigen presenting cells (Floege et al. 2021). This also suggests FMT might regulate IL‐10 to alleviate inflammation in IgAN. Although FMT has shown great potential in the treatment of certain intestinal diseases, its application still faces limitations. Identifying specific microbial characteristics that cause diseases is one of the main challenges of microbiota therapy. Additionally, most research on microbiota therapy is conducted in animal models and lacks human trials.

Traditional Chinese Medicine Zhen Wu Tang (ZWT) has been widely applied in chronic kidney diseases. The bioactivities of ZWT include anti‐inflammatory, diuretic, and anti‐hyperlipidemic effects. It displays efficacy in relieving symptoms manifested in the form of edema, dysuria, and oliguria. IgAN rats given ZWT showed observable reduction of proteinuria and amelioration of renal function. A recent study further indicated that gut flora homeostasis could be perturbed by IgAN, while ZWT ameliorated the gut microbiota dysbiosis caused by IgAN in rats (Liu et al. 2018; Li et al. 2021). Shenyan Kangfu tablet (SYKFT) is widely used for chronic kidney disease and alleviates clinical symptoms (Chen et al. 2021). The treatment with SYKFT increased the abundance of phylum Firmicutes in diabetic kidney disease mouse models and decreased the abundance of phylum Bacteroidetes. It also down‐regulated proinflammatory factors such as NF‐κB, TNF‐α, and IL‐1β to alleviate inflammation.

## 5 Conclusions and Future Perspectives

IgAN is the most common primary glomerular disease worldwide, with a complex pathogenesis involving multiple factors, including dysbiosis of the intestinal microbiota, immune system abnormalities, and impaired intestinal barrier function. Although the “multi‐hit hypothesis” explains the steps of IgAN pathogenesis, its pathogeny is still unclear. Recent research illustrates that abnormal immune function of the intestinal mucosa and dysbiosis of the intestinal microbiota are involved in the occurrence and development of IgAN. The investigation of the gut microbiota of IgAN patients has become one of the new targets for studying the pathogenesis and related treatments of IgAN. This paper introduced the alterations in the gut microbiota of IgAN patients, dietary intervention, and research progress on treatment strategies. Specific gut microbiota, such as *Bacteroides* and *Bifidobacterium*, are significantly reduced in IgAN patients, and the absence of these microbiota may be associated with the severity of the disease. Moreover, the alterations of the gut microbiota affect the metabolites, resulting in the improvement of clinical features. This is also observed in dietary interventions. A high‐fiber diet regulates gut microbiota composition, which is beneficial to relieve symptoms of IgAN. Novel therapy focuses on maintaining the intestinal microenvironment, such as FMT, which has been applied in clinical practice. The gut microbiota is expected to be a target for IgAN prevention, diagnosis, and treatment. Meanwhile, the safety and efficacy of the application of gut microbiota in IgAN need further exploration.

Future research is expected to elucidate how gut microbiota affects kidney function and how kidney health in turn affects the gut environment, providing a theoretical basis for developing new treatment strategies. Meanwhile, monitoring the dynamic changes of gut microbiota and even the metabolites during the development of IgAN and how these changes affect disease progression is helpful to understand the mechanisms of IgAN. Based on these researches, it is possible to develop personalized dietary and microbiome regulation strategies. Novel treatment strategies such as dietary interventions, probiotics, prebiotics, fecal microbiota transplantation, as well as Traditional Chinese Medicine have been evaluated as safe and efficacious in clinical trials. However, most current studies focus on short‐term efficacy. Evaluating the long‐term impacts of these interventions on IgAN and developing collaboration among multiple disciplines such as nutrition, microbiology, immunology, and nephrology are beneficial to provide more effective treatment and improve the quality of life of IgAN patients. Evaluating the long‐term impacts of these interventions on IgAN is beneficial to provide more effective treatment and improve the quality of life of IgAN patients. The future treatment of IgAN needs to pay more attention to personalization, and treatment plans will be developed based on the patient's genetic background, disease progression rate, and pathological characteristics. Meanwhile, in terms of dietary intervention, due to significant metabolic abnormalities in IgAN patients, personalized nutritional supplementation plans need to be provided for different patients. Multidisciplinary collaboration will become an important model for rehabilitation management, providing patients with comprehensive medical support and psychological care.

## Author Contributions

**Zhaoyang Dong:** visualization (lead), writing – original draft (lead), writing – review and editing (equal). **Ran Zhang:** writing – review and editing (equal). **Liang Shen:** writing – review and editing (equal). **Hong‐Fang Ji:** writing – review and editing (equal). **Haidong He:** writing – review and editing (equal). **Xiaoguo Ji:** conceptualization (supporting), resources (supporting), writing – review and editing (equal). **Liming Zhao:** funding acquisition (lead), project administration (lead), resources (equal), writing – review and editing (equal).

## Conflicts of Interest

The authors declare no conflicts of interest.

## Data Availability

The data that support the findings of this study are available on request from the corresponding author.
